# Synthesis of Candida Antarctica Lipase B (CALB) enzyme-powered magnetite nanomotor based on PCL/Chitosan Janus nanostructure

**DOI:** 10.1038/s41598-022-16777-0

**Published:** 2022-07-26

**Authors:** Fariba Mafakheri, Sepideh Khoee

**Affiliations:** Polymer Laboratory, School of Chemistry, College of Science, University of Tehran, Tehran, 14155-6455 Iran

**Keywords:** Polymer characterization, Polymer synthesis

## Abstract

In this work, we report the design and synthesis of internal energy-driven Janus nanomotors (JNMs), which are composed of certain reactive materials that are capable of converting chemical energy in the backbone of nanomotors into kinetic energy. For this purpose, superparamagnetic iron oxide nanoparticles (SPIONs) with the anisotropic surface were obtained via a Pickering emulsion. Modified chitosan (as hydrophilic polymer) and functionalized polycaprolactone (as hydrophobic domain) were covalently linked to the surface of bi-functional SPIONs to produce Janus nanoparticles (JNPs). Then, the CALB enzyme was loaded in the PCL hemisphere of JNPs to form the Janus nanomotor. When nanomotors are placed in the phosphate-buffered saline solution, the driving force for motion is provided by the decomposition of polyester into monomers and oligomers on one side of the JNMs. The trajectories of the nanomotors were recorded under different circumstances by a video microscope and analyzed by the mean squared displacement. The results show that the velocity of JNMs increases with an increasing percentage of the loaded enzyme. In addition, the diffusion coefficient enhances up to 87.67% in compared with nanoparticles without enzyme. Controlling the motion direction of JNMs by an external magnetic field is also possible, due to the presence of SPIONs.

## Introduction

Micro/nanomotors are miniature synthetic devices that can move through endogenous stimuli such as chemical fuels and catalysts^1^, or exogenous stimuli like electromagnetic or acoustic radiation forces in a liquid phase^2,3^. They can be designed to accomplish the desired moves and rotate, roll, shuttle, deliver or express other favorable behaviors based on particular stimuli. These motors are functionalized with biological or chemical reagents and used for various biomedical applications (such as drug delivery, microsurgery, or cell separation)^4^. Although the future of this research field can be bright, the utilization of biocompatible materials and fuels as the main challenge is still unresolved^5–8^. Most of the new strategies that tend to replace the metallic catalyst/H_2_O_2_ combination have not been successful, and new propulsion systems like H_2_ bubbles, generated from the reaction between a metal (Zn, Al, or Mg) and acid^9^ or H_2_O^10,11^, suffer from short lifetime and harsh reaction conditions which limit their utilization. Enzymes, as one of the most important biological molecules, have a critical role in the conversion of chemical energies into kinetic ones. Consequently, they can consider as the fuel for molecular machines because they use this kinetic energy and propel the carriers in biological systems. Some enzyme/fuel combinations are appropriate alternatives for nanomotors’ propulsion systems due to their high reaction rate^12^. Utilizing certain enzymes, with specific catalytic reactions, in making synthetic micro/nanoparticles such as carbon nanotubes^5^, carbon fibers, and microtubes^13^ can provide a bubble-production-based propulsion system that drives the nanoparticle via self-diffusion mechanism^14^. Enzymes like catalase^15^, glucose oxidase^15^, and urease^16,17^ are employed to conduct these processes; therefore, utilizing these non-toxic fuels to self-propel the micro/nanomotors can be the origin of an interesting idea. Based on this opinion, biocompatible polymers with specific functional groups that degrade with a particular enzyme can be good candidates for use in the synthesis of nanomotors’ shell. Polyesters are a class of synthetic polymers that have repeating ester bonds in their chains. Aliphatic polyesters can be degraded by enzyme-mediated hydrolysis to harmless products like carbon dioxide and water. Poly(ε-caprolactone) (PCL) is a polyester with a hydrolyzing rate from 3 months to 3 years depending on its molecular weight^18,19^, but embedding enzyme into the polymer chains can accelerate the degradation. Lipase enzymes, like Candida Antarctica Lipase B (CALB), can hydrolyze the ester bonds of aliphatic polyesters in aqueous media and has been recently used as a way to control the polymer lifetime^20,21^. Ester hydrolyzer enzyme CALB converts triglycerides into glycerol and fatty acids through lipolysis. This enzyme is from the hydrolases and is a subset of esterase^22^; however, in organic chemistry, lipases are used for the esterification of some molecules^21,23^.

Another major existing challenge related to nanomotors is the design of unique structures for easily preparation of a strong driving force. Over the past decades, micro/nanomotors (MNMs) with various geometries, including spherical, tubular, rod, etc., have been designed and fabricated, but most of these MNMs have Janus morphology^24,25^. Janus particles have anisotropic physical/chemical properties, and due to their unique pattern, they own hydrophilic characteristics on one side and hydrophobic ones on the other side of the particle.

A promising method for the synthesis of JNPs with two different faces is using a Pickering emulsion to immobilize nano/microparticles in a spherical substrate and then grafting a polymer on their remaining half surfaces, which produces Janus particles through the masking technique^26,27^. Janus magnetic SPIONs are considered optimistic candidates due to their excellent anisotropy in surface chemistry, shape, composition and non-toxic property^8,28,29^.

In this work, our target is to design and synthesize Janus nanomotors based on SPIONs that move by the CALB enzyme as bio-friendly fuel. Janus SPIONs are fabricated via the Pickering emulsion method, and their two sides are covered with polycaprolactone and chitosan separately. The CALB enzyme is loaded only in the PCL domain to produce an asymmetrical system. Thus, a self-decomposition nanomotor is constructed, in which the reactant and the products have no harmful effects on the human body. The motion of these nanomotors can be either self–propelled with self-diffusion mechanism or remotely controlled by an external force source such as a magnetic field.

## Experimental

### Materials

Ammonium hydroxide solution (25% NH_3_ in H_2_O), paraffin wax, 3-aminopropyl triethoxysilane (APTES), *ε-*caprolactone, triethylamine (TEA), Tin(II) 2-ethylhexanoate Sn(Oct)_2_, acryloyl chloride(AC), DL-malic acid, cisplatin, Candida Antarctica Lipase B (CALB, with 3.14 U/L lipase activity, (Supplementary Section S1.1)), sodium bis(2-ethylhexyl) sulfosuccinate (AOT), fluorescein, acridine orange, and dimethylformamide (DMF) were purchased from Merck Chemical Company. 1-Ethyl-3-(3-dimethylaminopropyl) carbodiimide (EDC), *N*-Hydroxysuccinimide (NHS) 98%, iron (II) chloride tetrahydrate (FeCl_2_(4H_2_O)) and iron (III) chloride hexahydrate(FeCl_3_(6H_2_O)) were obtained from Sigma-Aldrich. Chitosan oligosaccharide ($\overline{{M }_{n}}$= 3000 g/mol) was purchased from Golden-Shell Pharmaceutical Company (Ltd.), China. Ethanol, chloroform, and phosphate-buffered saline (PBS, pH 7.4) were supplied from Kian Kaveh, Iran.

### Methods

#### Synthesis of Janus nanoparticle

##### Decoration of half part of NH_2_-modified SPION with cisplatin (CisPt-Fe_3_O_4_-wax)

Janus NH_2_-modified SPION (APTES-Fe_3_O_4_-wax) was prepared in three steps according to Supplementary Sections S1.2–S1.4: preparing SPION, entrapping half part of SPION in wax microparticles via Pickering emulsion method, and modifying the remained bare surface with APTES. In the next step, cisplatin (30 mg, 0.08 mmol) was dissolved in 30 mL water and added to a flask containing 3 g of APTES-Fe_3_O_4_-wax dispersed in 30 mL of water. NaHCO_3_ (13 mg) was added to the flask and let to stir for 72 h. The produced CisPt-Fe_3_O_4_-wax was collected using an external magnetic field and washed with pure ethanol to remove excess cisplatin. The collected product was kept in the ethyl alcohol solvent.

##### Linking chitosan to half-surface of SPION via cisplatin (CS-CisPt-Fe_3_O_4_-wax)

A solution of 20 mg chitosan in 3 mL of water was added to a flask containing the dispersed mixture of CisPt-Fe_3_O_4_-wax (1 g) in ethanol (50 mL). The mixture was stirred at a stirring rate of 700 rpm at room temperature for 72 h. The prepared nanoparticles were collected by an external magnetic field and washed with pure ethanol and distilled water several times to remove the unreacted chitosan. The prepared CS-cisplatin-Fe_3_O_4_ was kept in ethyl alcohol for further use.

##### Removing the wax from the CS-CisPt-Fe_3_O_4_-Wax nanoparticles

Taking out the chitosan-modified SPIONs nanoparticles from wax microparticles was done according to the previously reported method^27^. The CS-CisPt-Fe_3_O_4_ nanoparticles were extracted from the wax microsphere by diluting the nanoparticle-containing wax balls in an excess amount of chloroform through the “bain-marie” technique at 100 °C*.* Chloroform solves the wax and separates the nanoparticles from the wax microsphere. Released nanoparticles can be collected using an external magnetic field. Finally, the Janus CS-cisplatin-Fe_3_O_4_ nanoparticles were washed with chloroform several times and kept in ethyl alcohol for the following usage.

##### Synthesis of CS-Fe_3_O_4_-PCL Janus nanoparticles

To synthesize the final Chitosan/PCL Janus nanoparticles, at first, acrylated poly(ε-caprolactone) (APCL) was prepared through a two-step method as introduced in the supporting information (ESI, Supplementary Sections S1.5 and S1.6). Following, synthesized APCL reacted with APTES according to the following procedure: APCL (15.8 g, 2.0 mmol) and APTES (0.45 mL, 2.0 mmol) were dissolved in anhydrous DMF and the solution was stirred at room temperature for three days. The reaction solution was then poured into the cold water to precipitate the triethoxysilane-functionalized poly(ε-caprolactone) (APAPTS) and was filtered. The precipitate was dried in a vacuum oven at 40 °C overnight and kept in a tightly sealed bottle for further use.

In the next step, 15.8 g APAPTS (2.0 mmol) was dissolved in anhydrous DMF, and the solution was stirred at room temperature for 30 min (solution 1). The previously prepared CS-CisPt-Fe_3_O_4_ (1 g) was dispersed in DMF (25 mL) under ultrasonic irradiation for 5 min and added to solution 1, rapidly. Stirring continued at room temperature for 72 h. The resulting CS-Fe_3_O_4_-PCL was separated using an external magnetic field and washed five times with chloroform, three times with ethanol, and kept in water.

### Preparation of the Janus nanomotors

#### Loading enzyme into the PCL chains of Janus nanomotor

- *Evaluation of the degradation effect of CALB on the PCL homopolymer* For this purpose, AOT was used as the surfactant to conjugate CALB to PCL. To load the enzyme in polymer, a solution of 3 mg polymer in 1 mL DCM was prepared, and then 0.933 mg AOT (2.0 mmol) and 0.3 mg CALB (10%w/w) were added to the solution. The mixture was stirred for 1 h, and afterward, the solvent was removed under reduced pressure. The PCL degradation rate was determined via ^1^H-NMR and UV-Vis spectroscopy. To evaluate the enzymatic degradation of PCL, five samples containing 5 mg polymer and 0.5 mg enzyme were added to 10 mL PBS at 37 °C and stirred for different times (12 h, 1 day, 2 days, 3 days, and 4 days). All samples were centrifuged after the determined lag times, the supernatants were separated and dried at room temperature, and the PCL degradation rate was analyzed by ^1^H-NMR. Similarly, 2 mg of PCL/CALB (10%w/w) and 1 mL PBS (pH = 7.4) were poured into a dialysis bag; then, the dialysis bag was immersed in 10 mL PBS and stirred at a rotating speed of 100 rpm. In the determined intervals, 10 mL of the solution containing released products was removed and replaced with the fresh PBS.
- *Preparation of nanomotors* 0.933 mg AOT (2 mmol) and CALB (10%w/w) were added to a dispersion of CS-Fe_3_O_4_-PCL Janus nanoparticles in anhydrous DMF (10% w/v). The mixture was vortexed for 1 h, and the enzyme-loaded nanoparticles were collected using an external magnetic field. Enzyme-loaded Janus nanoparticles were washed five times with ethanol to remove the unloaded enzyme and surfactant and kept in ethanol*.* To evaluate the motion of nanomotors, 5 mg of enzyme-loaded JNPs was dispersed in 5 mL of PBS (pH 7.4), and then transferred to a Petri dish to observe their motion behavior with Motic AE31 video microscope.

### Characterization techniques

^1^H NMR spectra were obtained by Bruker (Germany) DRX 500 spectrometer (500 MHz) using CDCl_3_ as the solvent. Synthesized poly(ε-caprolactone) and acrylated poly(ε-caprolactone) (PCL, APCL) were characterized using FT-IR spectroscopy (Bruker-Equinox55) from KBr pellets, and proton nuclear magnetic resonance (^1^HNMR) (Bruker, 500 MHz) using CDCl_3_ as the solvent. PCL degradation in the presence of enzyme was also confirmed via ^1^HNMR spectroscopy and UV–Vis spectroscopy. The attachment of cisplatin to APTES-Fe_3_O_4_-wax microspheres was evaluated using energy dispersive X-ray analysis (EDX) (Bruker X-Flash spectrometer). The weight average molecular weight ($\overline{{\mathrm{M} }_{\mathrm{w}}}$), number-average molecular weight ($\overline{{\mathrm{M} }_{\mathrm{n}}}$), and polydispersity index (PDI) of PCL were determined using the gel permeation chromatography (GPC) (PL-EMD 950) with 0.7 mL/min flow rate in tetrahydrofuran at 25 °C. The monodisperse polystyrene standards were used for calibration. The percentage of the attached polymers on the surface of the nanoparticles was determined by thermal gravimetric analysis (TGA) (TGA Q50 V6.3 Build 189) within the temperature range between 25 and 600 °C at the rate of 10 °C/min under nitrogen atmosphere. The size and shape of the prepared Janus nanoparticles were characterized by scanning electron microscopy (HITACHI S 4160, accelerating voltage = 10.0 kV, vacuum levels: 10^–7^ Pa in electron gun chamber, and 10^–4^ Pa in specimen chamber, and magnification = 120KX) and transmission electron microscopy (TEM) (Tecnai G2 F20 S-Twin, accelerating voltage = 200 kV, Vacuum levels: 10^–5^ (Pa) in specimen chamber and 10^–6^ (Pa) in electron gun chamber, and magnification = 21X-700KX). The magnetic properties of Fe_3_O_4_ NPs and CS-Fe_3_O_4_-PCL JNPs were measured by using a Vibrating Sample Magnetometer (VSM-MDKB, Magnetic Daneshpajoh Kashan, Iran). The morphology of the masked NPs in wax were recorded using a Motic AE 31 optical microscope. The prepared nanoparticles were loaded with fluorescein (as hydrophobic dye) and acridine orange (as hydrophilic dye), to distinguish each domain in Janus nanomotors. For this purpose, 3 mg of the prepared nanomotors were dispersed in 1 mL of acetone; and 1 mg of each dye (fluorescein and acridine orange) was added to the suspension. Three drops of DMSO were added to the mixture to increase the solubility of fluorescein in acetone and the mixture was stirred for 1 h. A few amounts of the suspension were dropped on a lamella, dried in an oven at 40 °C, and imaged with a fluorescence microscope (Zeiss, Axioskop 2 plus, Germany).

## Results and discussion

### Synthesis and characterization of Janus nanoparticles

**Scheme 1:**
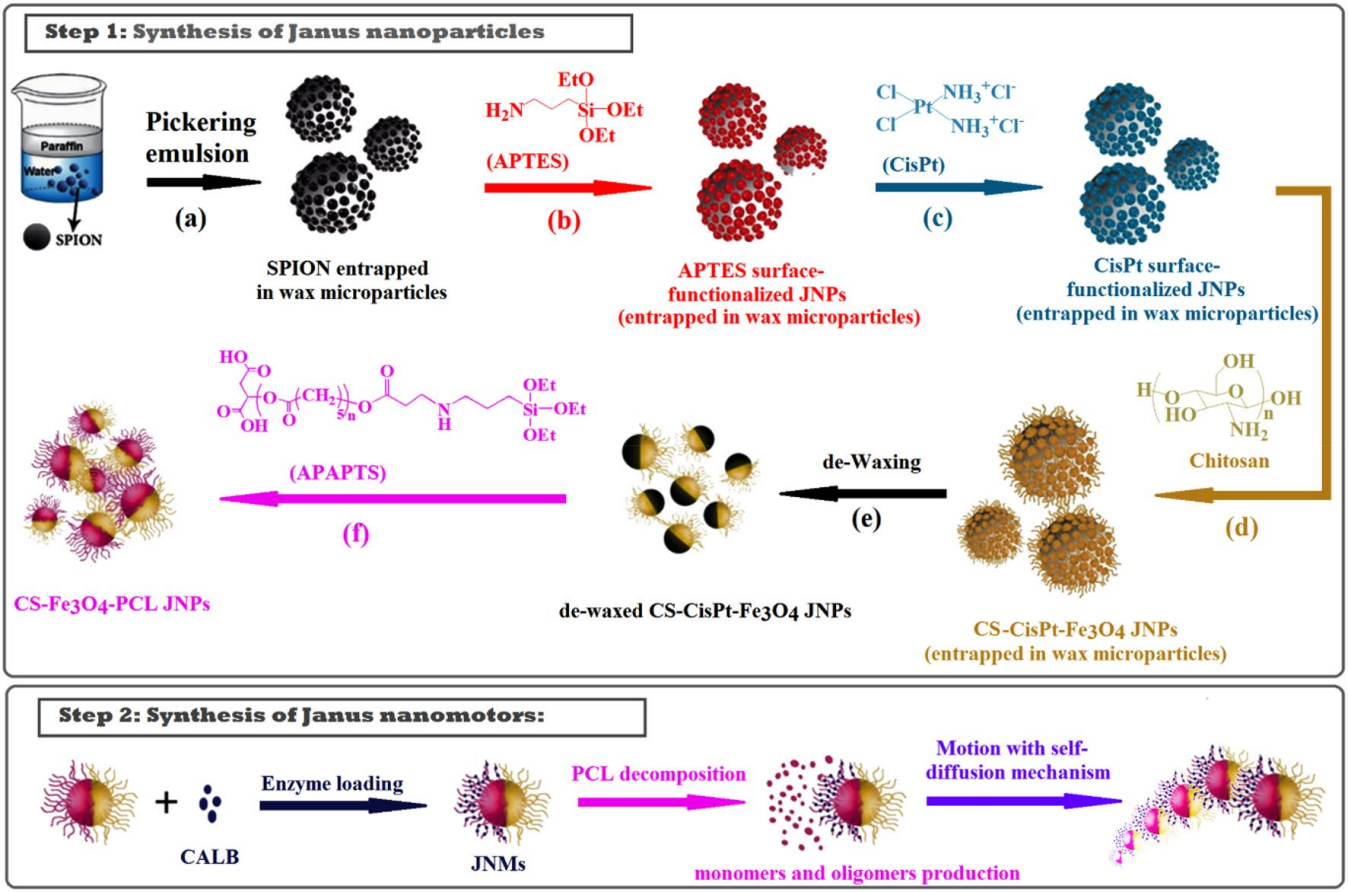
Step 1: Schematic presentation of (CS-Fe_3_O_4_-PCL) Janus nanoparticle synthesis: entrapping half part of SPION in wax microparticles via Pickering emulsion method (**a**), modifying the remained bare surface of SPIONs with APTES (**b**), substitution reaction of the Cisplatin chloride groups by APTES amine groups (**c**)*,* substitution reaction of the Cisplatin remained chloride groups by chitosan (**d**), extraction of CS-CisPt-Fe_3_O_4_ JNPs from wax microspheres by de-waxing (**e**), preparation of CS-Fe_3_O_4_-PCL JNPs by conjugation of the hydrophobic polymer (APAPTS) on the free surface of JNPs (**f**); Step 2: Synthesis of Janus nanomotors: Enzyme-loading on the PCL section of JNPs and motion of JNMs by self-diffusion mechanism.

Here, we present a general approach adopted from the Pickering emulsion method for Janus nanomotors fabrication (Scheme 1). Through the high-speed stirring of a paraffin and water mixture containing Fe_3_O_4_ nanoparticles, paraffin micro-droplets were produced in oil/water emulsion, which was stabilized by Fe_3_O_4_ nanoparticles that play a surfactant role. Subsequently, by functionalizing one side of these nanoparticles with chitosan, Janus nanoparticles were synthesized. The amphiphilic Janus nanoparticles were obtained after removing the wax microparticles and adding PCL. By loading the enzyme onto one side of the particles, we obtained Janus nanomotors, which can be driven via asymmetric degradation of poly-caprolactone.

**Figure 1:**
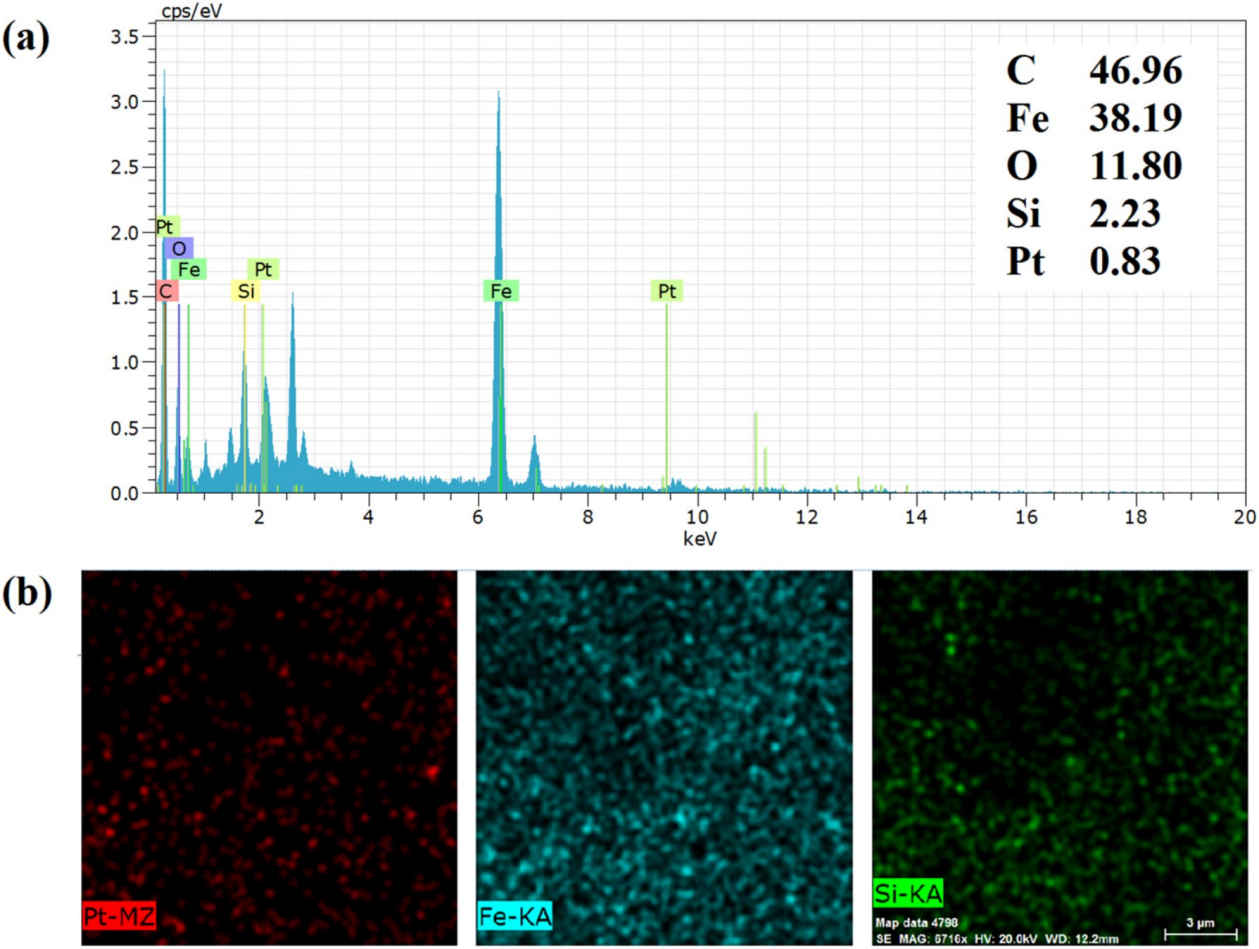
Energy-dispersive X-ray (EDX) spectrum (**a**) and elemental mapping: Electron micrograph region (**b**) of CisPt-APTES-Fe_3_O_4_-wax microparticles.

Accordingly, as the first step, SPIONs were synthesized and trapped in wax microspheres, and the free face of nanoparticles was modified with APTES^27^ (Supplementary Scheme S1). Janus aminated-SPIONs (NH_2_-Fe_3_O_4_-wax particles) were washed by chloroform to remove wax microparticles and then analyzed by FT-IR spectroscopy. FT-IR spectrum of NH_2_–Fe_3_O_4_ Janus nanoparticles is shown in Supplementary Fig. S1. The strong N–H and O–H absorption bands at 3400 cm^−1^, two peaks at 1030 and 1118 cm^−1^ related to the Si–O stretching, and two absorption bands with maxima at 1100 and 1600 cm^−1^ attributed to C–N stretching and N–H bending vibrations of amino groups of the APTES prove the modification of the Janus SPIONs. In the next step, chitosan must be attached to the bare surface of SPIONs. Cisplatin was selected as a linker to conjugate chitosan to the surface of SPIONs. For this purpose, cisplatin was reacted with the remained amine groups of APTES via the nucleophilic substitution reaction between the chloride ligand of cisplatin and the amine groups of APTES (Supplementary Scheme S2, Step 1). Energy-dispersive X-ray spectroscopy (EDX) was used to identify the chemical composition of CisPt-Fe_3_O_4_-wax microparticles. The EDX spectrum approved the presence of expected elements in the nanoparticle such as C, Fe, O, Si, and Pt with the weight percent of 46.96, 38.19, 11.80, 2.23, and 0.83 wt%, respectively (Fig. 1a). The higher amount of C was due to the presence of carbon-rich wax. Also EDX mapping (Fig. 1b) showed that Pt and Si atoms were uniformly dispersed on the surface of the CisPt-Fe_3_O_4_-wax sample. The optical microscope image of CisPt-Fe_3_O_4_-wax microparticles revealed a mean diameter of 3.7 ± 0.48 µm (Supplementary Fig. S2).

**Figure 2:**
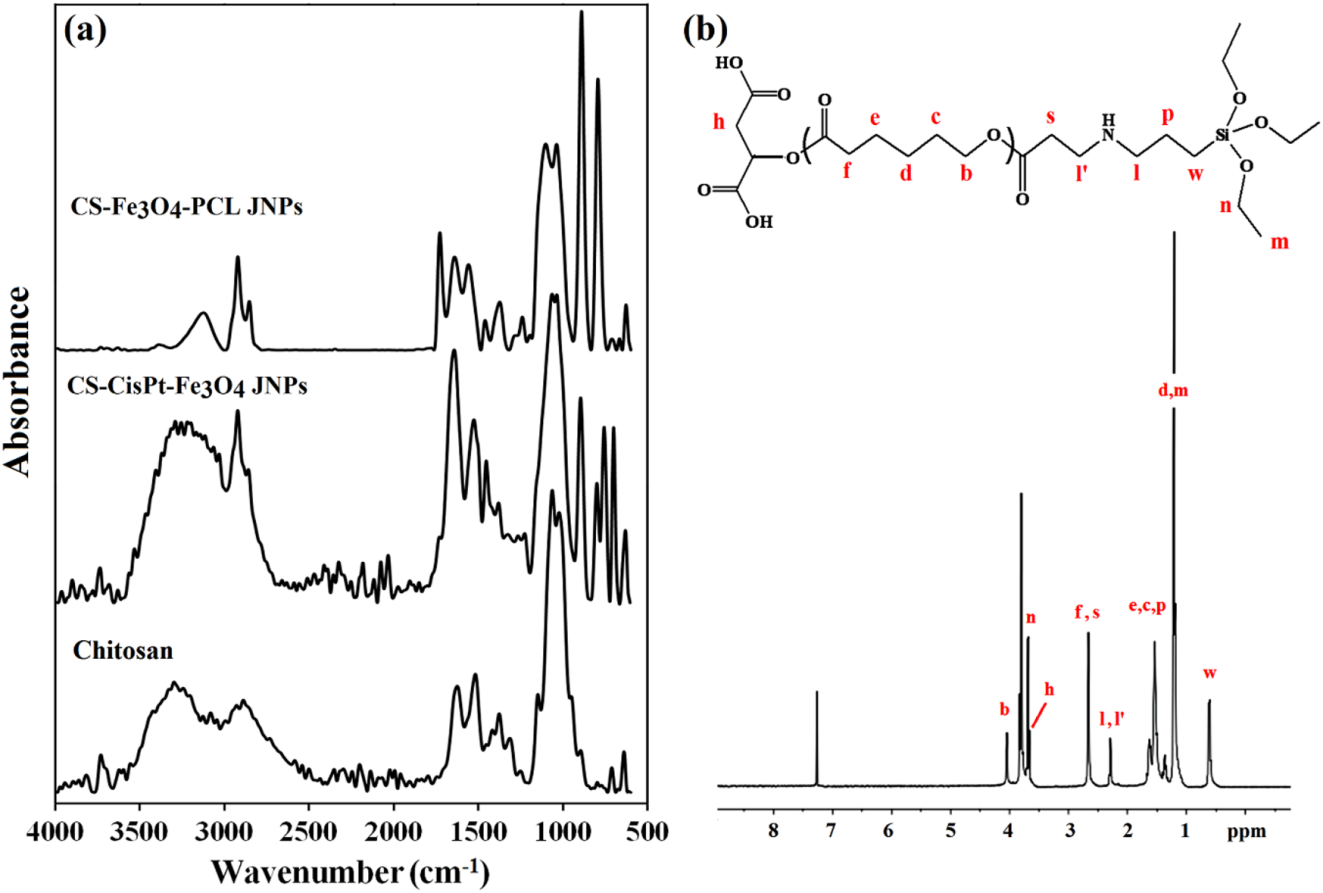
FT-IR spectra of chitosan, Janus (CS-CisPt-Fe_3_O_4_) nanoparticles, and final Janus (CS-Fe_3_O_4_–PCL) nanoparticle (**a**) and 1H-NMR spectrum of APAPTS.

Similarly, the remaining chloride groups of CisPt were replaced with Cs via the ligand exchange method (Supplementary Scheme S2, Step 2). The wax-free CS-CisPt-Fe_3_O_4_ nanoparticles were analyzed by FT-IR spectroscopy, and its result was compared with pure chitosan. As illustrated in Fig. 2a, a similar pattern was observed for both samples, but the characteristic peak of cisplatin that appeared at 885 cm^−1^ confirms the formation of CS-CisPt-Fe_3_O_4_ Janus nanoparticles.

In our design, PCL constructed the hydrophobic domain of Janus nanoparticles. To prepare this domain, ɛ-caprolactone was polymerized through the ring-opening polymerization using malic acid as the initiator^30^ (Supplementary Scheme S3). In ^1^H-NMR, the methylenic protons of the PCL chains appeared as multiplets at δ = 4.05 (b), δ = 1.64 (c, e), δ = 1.37 (d), and δ = 2.30 (f) ppm (Supplementary Fig. S3). The shifts at δ = 3.64 (h) indicated the methyl group of initial d,l-malic acid. Accordingly, the molecular weight of PCL was calculated and found to be about 7900 g/mol. PCL was functionalized with acrylate groups using previous methods^30^ (Supplementary Scheme S4). The ^1^H-NMR spectrum of acrylated PCL illustrated all the characteristic peaks of PCL as well as three new peaks at δ = 5.8 (i), 6.11 (j), and 6.37 (k) ppm, corresponding to the hydrogens on the acrylate group (Supplementary Fig. S4). In the FT-IR spectrum of acrylated PCL (Supplementary Fig. S5), characteristic peaks of PCL appeared at 1721 cm^−1^ (C=O stretching bond), 1170 cm^−1^ (O–C–O esteric groups), and 2943.4 cm^−1^ (C–H stretching bond), and the new weak peak at 1648 cm^−1^ was attributed to C=C of acrylate groups. APTES modified APCL (APAPTS) was synthesized according to Supplementary Scheme S5 and characterized by ^1^H-NMR in CDCl_3_. The ^1^H-NMR spectrum of APAPTS elucidated all the characteristic peaks of PCL as well as the five new signals at 0.60 (w), 1.21(m), 1.54 (p), 2.29 (l), and 3.50 (n) ppm related to APTES (Fig. 2b). The peak at 2.66 ppm belonged to methylene protons attached to the carbonyl group (f, s).

**Figure 3:**
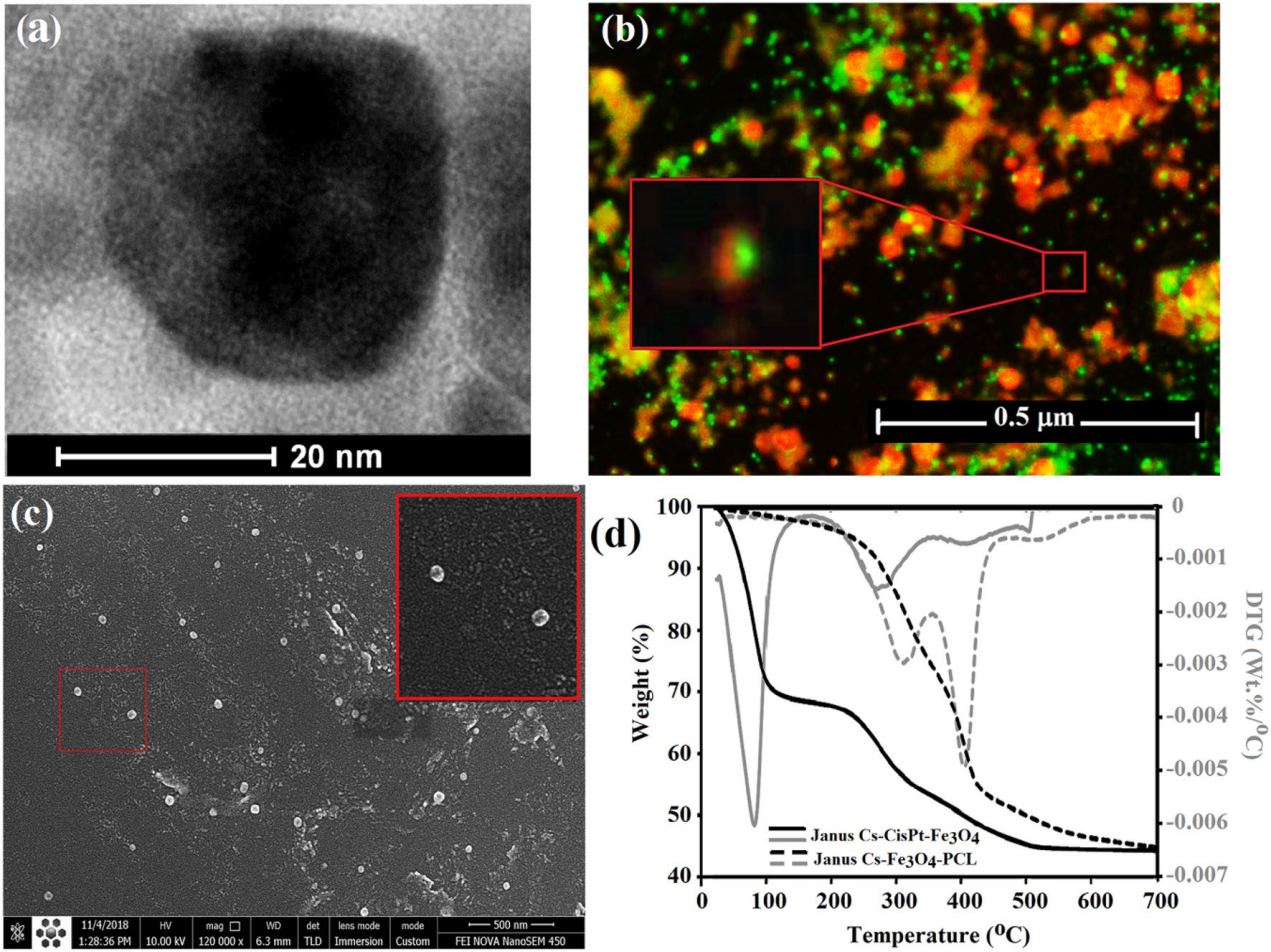
HRTEM image (**a**), Fluorescence microscopy image of fluorescein and acridine orange loaded Janus nanoparticles (**b**), SEM image (**c**), and TGA/DTG curves (**d**) of Janus CS-Fe_3_O_4_-PCL.

The other side of the CS-CisPt-Fe_3_O_4_ nanoparticles was modified with APAPTS (Supplementary Scheme S6). The reaction between the remained hydroxyl groups of Janus SPION and triethoxysilane functional groups on polycaprolactone resulted in preparing the Janus polymeric nanoparticles. FT-IR spectrum of CS-Fe_3_O_4_-PCL showed all characteristic peaks of chitosan and PCL domains (Fig. 2a). The HRTEM image of JNPs (Fig. 3a) showed spherical particles with two distinguishable sides. The synthesized JNPs exhibited a diameter below 40 nm. The morphology of Janus nanoparticles after loading fluorescein and acridine orange as hydrophobic and hydrophilic dyes was examined by fluorescence microscopy (Fig. 3b). The existence of two domains with red and green color in single particle, related to the replacement of hydrophilic and hydrophobic dyes prove that the CS and PCL domains are completely separate from each other. These confocal images confirmed that the synthesized nanoparticles had Janus morphology.

SEM images revealed that the morphology of JNPs was spherical, with a mean diameter of about 37 nm (Fig. 3c). Thermogravimetric (TGA) and derivative thermogravimetric (DTG) analyses were used to elucidate the thermal behavior of JNPs (Fig. 3d). In the TGA curve of CS-CisPt-Fe_3_O_4_, the first notable weight loss occurred at 100 °C owing to the evaporation of physically absorbed water. The second weight loss that started from 190 °C was related to the decomposition of conjugated chitosan^31^. The TGA curve of J(CS-Fe_3_O_4_-PCL) showed two peaks in DTG thermograms. The first one located in the 314 °C regions is related to the decomposition of the chitosan layer and determined a nearly 24% weight for the chitosan domain. The other weight loss with T_max_ = 406 °C attributed to the thermodegradation of the PCL layer presented on the surface of JNPs (Fig. 3d). According to this thermograph, the amount of PCL was calculated and found to be about 29%. Supplementary Table S1 summarizes the results of TGA and DTG analyses.

Superparamagnetism is a crucial property for using magnetic nanoparticles in biomedical and bioengineering fields. Nanoparticles with this property would not aggregate or redisperse rapidly after removing the magnetic field. The magnetization curves of bare Fe_3_O_4_ NPs and CS-Fe_3_O_4_–PCL JNPs were recorded with VSM and illustrated in Supplementary Fig. S6. The saturation magnetization (Ms) value of bare Fe_3_O_4_ NPs decreased from 32.58 to 21.03 emu/g by covering magnetite nanoparticles with chitosan/PCL layers. However, this amount of saturation magnetization is sufficient for various biological applications. As well as, the magnetization hysteresis was close to zero for both samples.

### Refueling the nanomotors and evaluating their motion

In this project, JNMs propulsion may happen via two mechanisms: (i) through diffusiophoresis propulsion that occurred by decomposition of polycaprolactone to their monomers and oligomers (known as self-propulsion); and (ii) by an external magnetic field.

**Figure 4:**
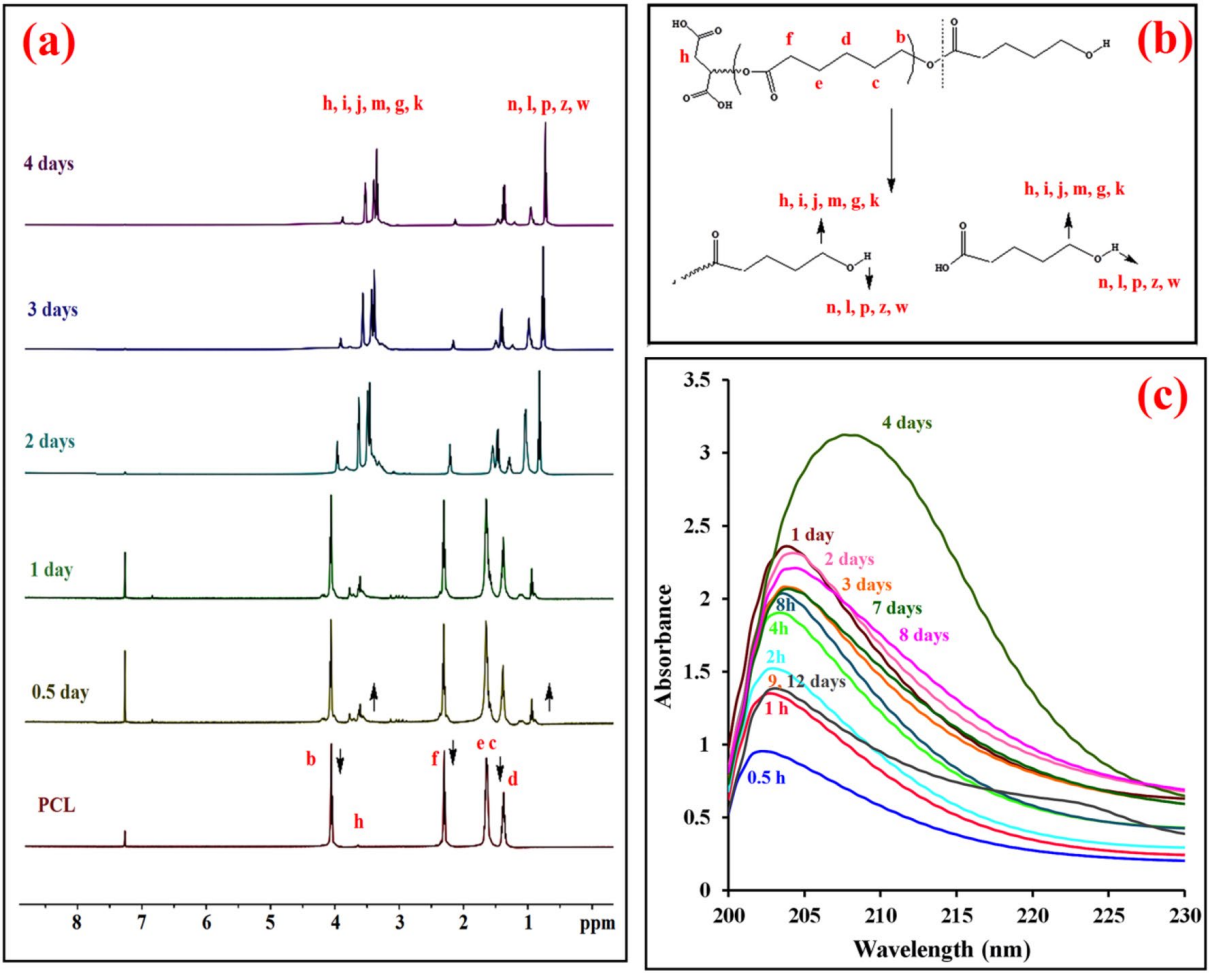
(**a**) ^1^H NMR spectrum of PCL before and after 12 h, 1, 2, 3, and 4 days degradation, (**b**) the proposed mechanism for PCL degradation, and (**c**) UV–Vis absorption spectra of the degradation of PCL containing the enzyme after immersion in PBS at different times (π → π* transition region).

Self-diffusiophoresis is a propulsion phenomenon in which the movement of particles is induced by a concentration gradient of the reaction products. This propulsion mechanism is more commonly employed in spherical Janus micro-/nanomotors. In this system, the catalyst (CALB) is loaded on one side of the micro-/nanomotor; the reaction products (monomers and oligomers) preferentially accumulate wherein the catalyst exists. Hence, a concentration gradient is produced along the surface of the micro-/nanomotor. As the reaction products reach a certain point, the local concentration becomes higher, and the degradation products start to diffuse away from the catalyst, which in turn produces a force leading to the movement of the micro-/nanomotor. In an ideal case, the degradation of the polymer should reach a critical level then the nanomotor starts moving due to the diffusion mechanism. Since the complete degradation of PCL can take several years under the normal condition at neutral pH^21^, CALB was used to perform the hydrolysis reaction faster. The progression of changes in PCL molecular weight was determined by ^1^H-NMR. Five samples of 10% CALB-embedded PCL poured into 10 mL PBS were kept at 37 °C for 12 h, 1-day, 2-day, 3-day, and 4-day intervals. The methylenic protons of the PCL chains in CDCl_3_ were observed as multiplets at δ: 4.00 (b), 2.3 (f), 1.30 (d), and 1.55 (c, e) ppm (Fig. 4a). Over time, the intensity of the PCL peaks decreases indicated that polyester degradation occurred in the presence of the enzyme. The appearance of new signals in the 3.30–4.11 and 0.74–1.43 ppm regions related to the protons of methylenic groups adjacent to the terminal hydroxyl groups and the OH group^32^, respectively, prove the production of alcoholic compounds with different chain lengths (Fig. 4b).

**Figure 5:**
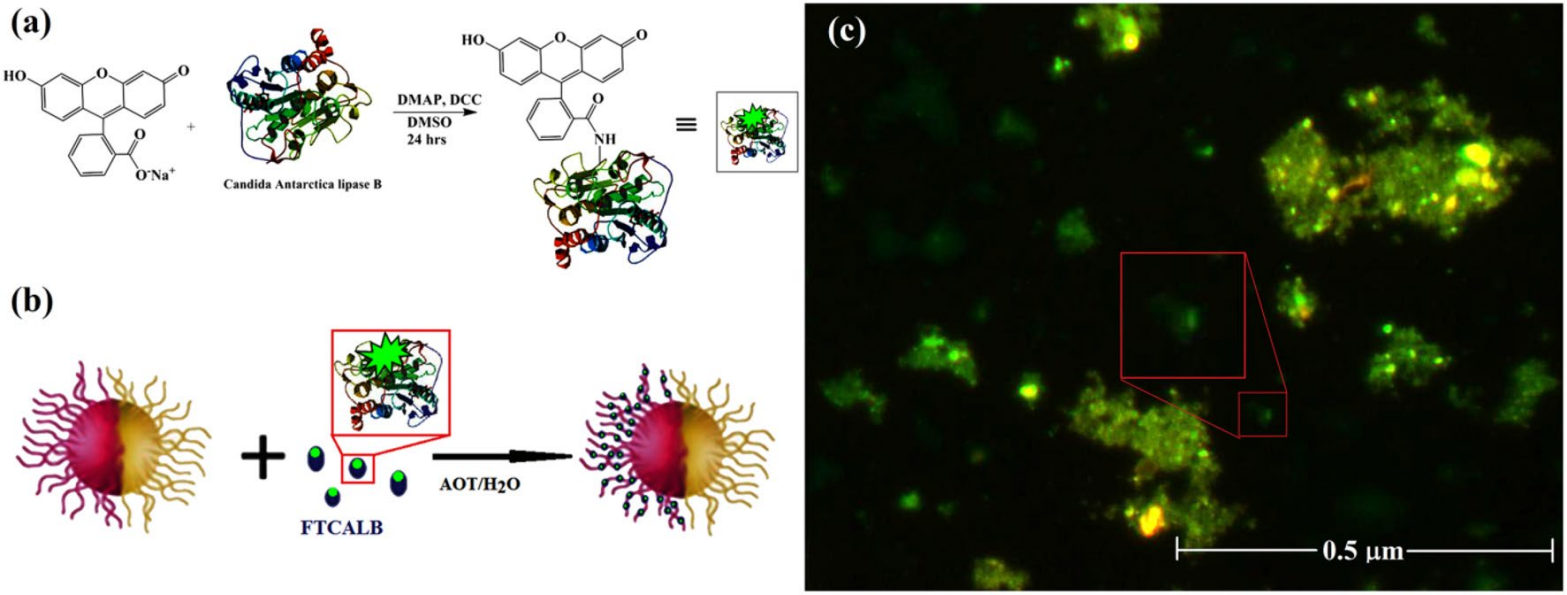
Proposed mechanism for the attachment of CALB to Fluorescein (**a**), Schematic illustration of the preparation of fluorescent Janus nanoparticles from physical entrapment of FTCALB in the JHPs (**b**), and Fluorescence microscopy image of fluorescein-tagged enzyme loaded in the half surface of the Janus nanoparticles.

UV–vis spectroscopy was employed to gain further insight into the capability of CALB in PCL degradation (Fig. 4c). The filtrate from degradation media demonstrates a peak around 205 nm related to the creation of hydroxyl carboxylic acid fragments^33^, which increase by increasing the incubation time. The effect of the CALB enzyme on the degradation of the PCL domain of JNPs was examined by measuring the ability of JNPs to self- propel after refueling with the enzyme. For this purpose, at first, the enzyme was loaded in CS-Fe_3_O_4_-PCL Janus nanoparticles using AOT as a surfactant. We predicted that the enzyme occupied only one-half of JNPs. To verify the site-selective physical attachment of CALB on the PCL domain of JNPs, CALB was coupled to the fluorescein (FTCALB) (Fig. 5a). After the Incubation of JNPs with fluorescein-tagged protein (FTCALB) (Fig. 5b), the fluorescence microscope was used to confirm the presence of the modified protein on the hydrophobic hemisphere of the nanoparticles. Embedding the respective proteins to one side of JNPs resulted in the adsorption of the fluorescein on one side of the beads. The fluorescent image in Fig. 5c shows the evidence for the presence of protein on the half of the JNPs surface through fluorescent protein staining.

The self-propulsion of the nanomotors was investigated by optical microscopy camera, and analyzed by the trajectories obtained from tracking the motion of nanoparticles. The self-propulsion of JNMs was systematically analyzed in different enzyme concentrations and at various contact times. Mean squared displacements (MSD) were calculated based on developed MATLAB codes, as well as, the speed and diffusion coefficient parameters were obtained for all particular trajectories. The impact of CALB enzyme in bio-catalytically driven motion of the JNPs was first tested for the CALB-free Janus nanoparticles. The enzyme-free Janus nanoparticle was suspended in PBS media as the motion fuel, and its motion was recorded using a digital video camera (Supplementary Video S1, ESI†). The motion tracking of Janus nanoparticles in PBS (pH 7.4) at a contact time of 30 min is obtained from the recorded video. The enzyme-free nanoparticles did not have significant movement in the phosphate buffer media. The results obtained from the trajectory of JNPs were used to plot the MSD as a function of time and calculate their average diffusion coefficient according to Eq. (1)^34^:

1 $$De=\frac{MSD}{4\Delta t}.$$

**Figure 6:**
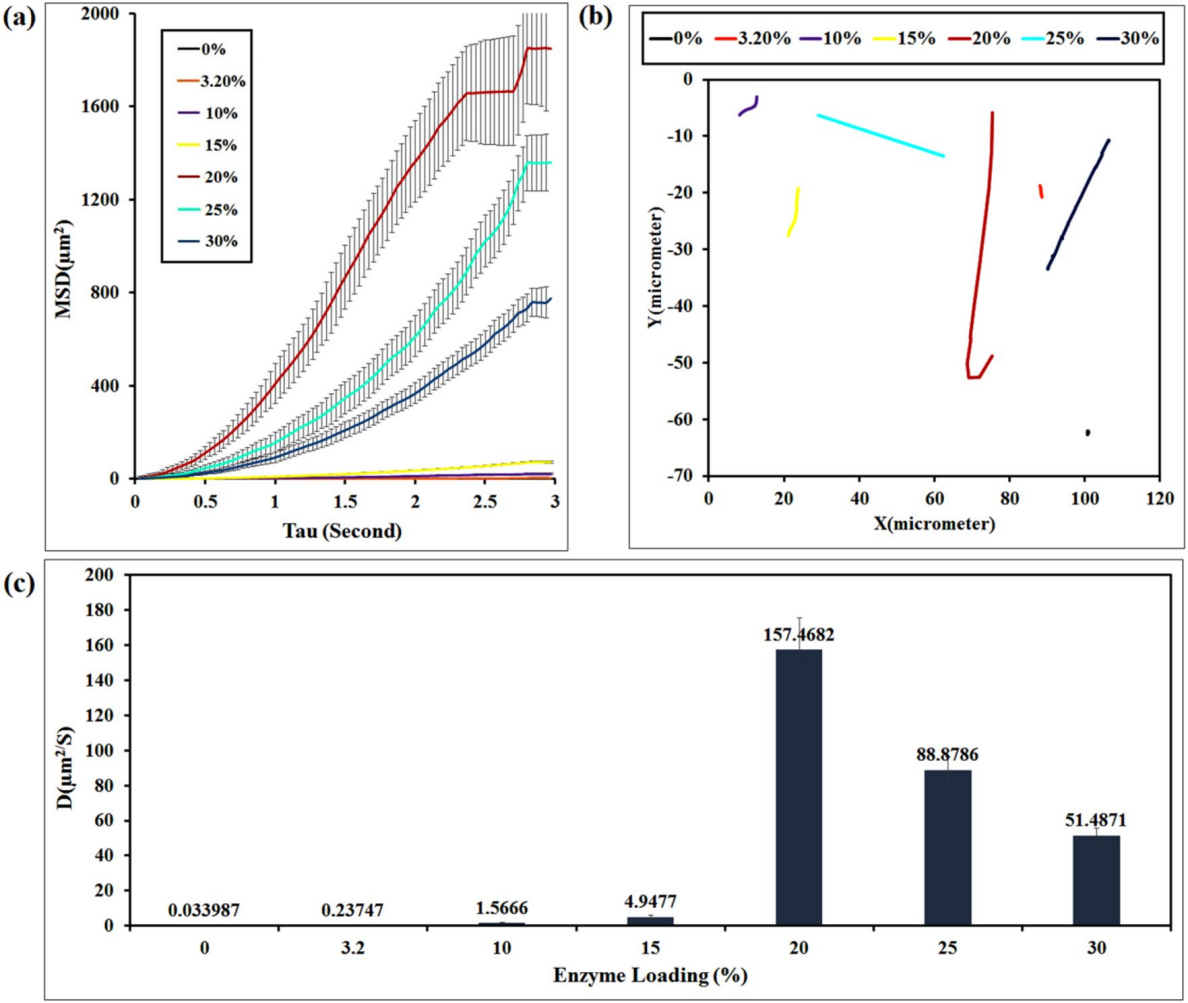
(**a**) Distributions of MSD values obtained for individual JNPs at the time scale Δt = 3 s for different percentages of enzymes loaded on PCL chains (3.2%, 10%, 15%, 20%, 25%, and 30% w/w; (**b**) typical single-particle tracking trajectories of JNMs, (**c**) distributions of diffusion coefficient (D) values obtained for individual JNPs at the time scale Δt = 3 s (obtained from Supplementary Video S2–S7 in the ESI†).

The diffusion coefficient of the Brownian motion (without CALB) calculated from the recorded video was 0.033987 ± 0.00154 μm^2^/s. To evaluate the ability of enzyme to generate the desired force and to propel the Janus nanomotor, CALB was physically immobilized onto the PCL side of the JNPs using sodium bis (2-ethylhexyl) sulfosuccinate (AOT) as the surfactant (Scheme 1, Step 2). The motion of (PCL-Fe_3_O_4_-CS) JNPs loaded with 3.2, 10, 15, 20, 25, and 30% (w/w) of CALB enzyme was investigated in the PBS solution, and the corresponding videos are included in the Supplementary Videos S2–S7. By degradation of polycaprolactone with the CALB enzyme, PCL can decompose to its monomers and oligomers. We predict that increasing the percentage of the loaded enzyme could be more effective for this degradation process. CALB-modified micromotors showed a significant increase in self-propulsion at higher enzyme concentrations. As expected, the enzyme-loaded Janus nanomotors moved in a straight pathway via the self-diffusion propulsion mechanism. It seems that chitosan acts as the head of the nanomotor and the degrading PCL as its tail. Colored lines in each movie indicate the diffusion path of the Janus nanomotors. The plot of the individual average MSD versus Δt for each tracked JNMs demonstrated that by increasing the amount of enzyme from 3.2 to 20% (w/w), an increase in the slope of MSD curves was observed. With increasing enzyme from 20 to 30% (w/w), the enzymatic degradation rate of polymer chain decreased dramatically (Fig. 6a), which is due to the saturation of nanoparticles with enzyme^35^. The typical trajectories of the JNMs with different amounts of enzyme presented the same behavior (Fig. 6b). Similarly, the velocity and diffusion coefficient parameters of JNMs’ motion increased from 0.63544 ± 0.0258 to 14.48 ± 0.0467 μm/s, and 0.23747 ± 0.0175 to 157.4682 ± 0.0368 μm^2^/s, respectively (Fig. 6c).

**Figure 7:**
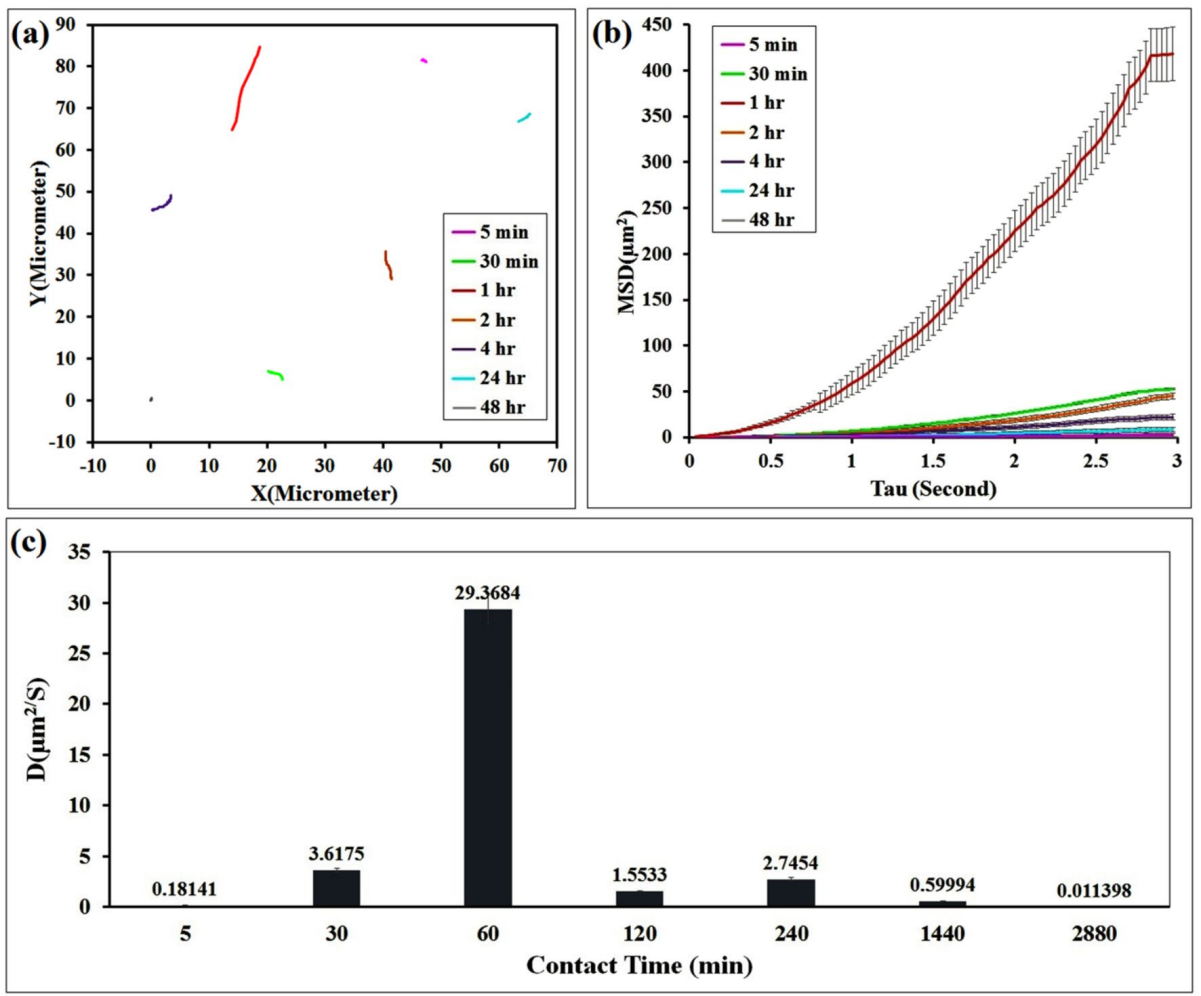
(**a**) Distributions of MSD values obtained for the self-propelled catalytic JNMs in PBS (pH = 7.4) with CALB loading (10% w/w) on the surface of JNPs at different contact times, (5 min, 30 min, 1 h, 2 h, 4 h, 24 h and 48 h), (**b**) distributions of diffusion coefficient (D) values obtained for individual JNMs (Supplementary Video S8–S14 in the ESI†).

Enzymatic activity is one of the essential parameters for moving JNPs. The concentration gradient around each nanoparticle must reach a critical level to provide an appropriate driving force for starting the propulsion of the nanomotors via the diffusion mechanism. Therefore, to assess the needed enzyme functioning time for the movement of the nanomotors, JNPs were loaded with 10% CALB enzyme, and the videos were recorded after different contact times (5 min, 30 min, 1, 2, 4, 24, and 48 h) (Supplementary Video S8–S14 in the ESI†). Figure 7a,b exhibited the trajectories of JNMs and the related MSD analysis at different contact times, respectively. After placing the nanomotor in PBS media for five minutes, this time was not sufficient for the enzyme to degrade PCL. Consequently, the expected concentration gradient needed for the movement of the nanomotors was not provided, and the JNMS propel at low speeds. As a result, Fig. 7c illustrates that the diffusion coefficient and velocity parameters of JNMs’ motion increased from 0.18141 ± 0.10713 to 29.3684 ± 0.1301 μm^2^/s and 0.51025 ± 0.15433 to 6.8874 ± 0.38124 μm/s by increasing the contact time from 5 min to 1 h. The measured MSDs show that the diffusion coefficient parameters of JNMs’ motion decreased from 29.3684 ± 0.1301 to 0.59994 ± 0.058965 μm^2^/s by increasing time from 1 to 24 h. Over time, after 48 h, the nanomotors are nearly inactive and do not show any movement. The reason for this particle behavior can be explained by reducing enzymatic activity or depletion of the polyester substrate.

It is expected that the nanoparticles constructed from SPIONs would be affected by the external magnetic field. To evaluate this phenomenon, an external magnetic field is applied to the Petri dishes containing JNMs with 10% CALB, and its position changed over time. Nonetheless, all samples followed the magnetic field, no aggregation was observed. Typically, in Supplementary Video S15, the position of the external magnet was changed twice from right to left and left to right, and finally, it was settled at the bottom of the Petri dish. As shown in the video, by applying the external magnet bar, all nanoparticles start following the direction of magnet bar with a very high velocity compared to the no-field mode.

## Conclusions

We successfully fabricated enzyme-driven Janus nanomotors based on PCL/Chitosan JNPs via the Pickering emulsion. The physicochemical properties of the prepared Janus nanoparticles were investigated by FT-IR and ^1^H-NMR spectroscopy, thermogravimetric analysis (TGA), scanning electron microscopy (SEM), and transmission electron microscopy (TEM). To investigate the enzymatic degradation of the PCL domain of JNPs, CALB was loaded into polycaprolactone homopolymer and then the catalytic degradation of PCL by this enzyme in PBS media was confirmed by ^1^H-NMR and UV–visible spectroscopy. Similarly, CALB was loaded in chitosan/PCL Janus nanoparticles using AOT as a surfactant. The biocatalytic reaction between lipase B (CALB) and the polyester substrate (PCL) provides the appropriate power for the movement of JNPs. The motion of the Janus nanomotors was tracked by optical microscopy and calculated from MSD analysis. The movement of catalytic Janus nanomotors in the presence of different percentages of the loaded enzyme [3.2, 10, 15, 20%, 25%, and 30% (w/w)] in PBS solution was investigated by the single-particle tracking method. The MSD calculations show that by increasing the percentage of loading enzyme above 20%, the diffusion coefficient and the velocity decrease due to the increase in viscosity and substrate inhibition. Due to the decrease in enzyme activity over time, the diffusion coefficient and velocity values of JNMs were also evaluated at different contact times (5 and 30 min, 1, 2, 4, 24, 48 h). The diffusion coefficient of JNMs with a contact time of 5 min shows a motion similar to the Brownian one, because the concentration gradient of monomers and oligomers from polycaprolactone degradation have not reached a critical level. The maximum velocity and diffusion coefficient values were obtained for the samples with a contact time of 1 h. It seems that the enzyme activity reached its maximum at this interval and the upper concentration gradient provided during this critical time. The evidence of this study shows the velocity and diffusion coefficient values decrease for a contact time of more than one hour, and these nanomotors do not exhibit any movement after 48 h due to reduced enzymatic activity or depletion of the polyester substrate. In addition, we found that the external magnetic field can change the motion direction of the synthesized JNMs.

## Supplementary Information

Supplementary Information.

Supplementary Video 1.

Supplementary Video 2.

Supplementary Video 3.

Supplementary Video 4.

Supplementary Video 5.

Supplementary Video 6.

Supplementary Video 7.

Supplementary Video 8.

Supplementary Video 9.

Supplementary Video 10.

Supplementary Video 11.

Supplementary Video 12.

Supplementary Video 13.

Supplementary Video 14.

Supplementary Video 15.

## Data Availability

All data generated or analyzed during this study are included in this published article (and its Supplementary Information files).
