# Supplementary material for: Synthesis of Candida Antarctica Lipase B (CALB) enzyme-powered magnetite nanomotor based on PCL/Chitosan Janus nanostructure
Source: Sci Rep. 2022 Jul 26;12:12758. doi: 10.1038/s41598-022-16777-0 (PMC9325781; doi:10.1038/s41598-022-16777-0)
Supplement: Supplementary file 1 — Supplementary Information. [file 41598_2022_16777_MOESM1_ESM.docx]

**Supporting Information**

**Synthesis of candida antarctica lipase B (CALB) enzyme-powered magnetite nanomotor based on PCL/Chitosan Janus nanostructure**

**Fariba Mafakheri, and Sepideh Khoee***

^a^ Polymer Laboratory, School of Chemistry, College of Science, University of Tehran, 14155-6455, Tehran, Iran

* Email: [khoee@khayam.ut.ac.ir](mailto:khoee@khayam.ut.ac.ir)

**Table of Contents**

# S1. Experimental Procedures

*S1.1. lipase activity assay*

*S1.2. Synthesis of Fe3O4 nanoparticles*

*S1.3. Preparation of Fe3O4-wax composite microsphere*

*S1.4. Modifying waxed SPIONs with APTES*

*S1.5. Synthesis of poly (ε-caprolactone) (PCL)*

*S1.6. Acrylation of PCL*

# S2. Additional Data

# S1. Experimental Procedures

*S1.1. lipase activity assay*

Lipase activity was performed in aqueous buffer as the method described by Secundo et al. [1]. The hydrolytic activity of purified CALB was determined using vinylacetate as substrate (100 μL/mL) in 0.05 M potassium phosphate buffer (pH 7) at 25°C. The produced acetic acid was titrated with sodium hydroxide in the presence of phenolphthalein as the indicator.

*S1.2. Synthesis of* Fe_3_O_4_ *nanoparticles*

Magnetite nanoparticles were synthesized via co-precipitation of Fe (II) and Fe (III) chloride in alkali solution according to the previously reported technique [2]. Brieﬂy, 1g of FeCl_2_.4H_2_O and 2.6 g of FeCl_3_.6H_2_O were dissolved in 25 mL distilled water under N_2_ purging and kept at 75 °C for 10 min, then 10 mL of ammonia solution (25%) was added into the prepared mixture dropwise under vigorous stirring and kept at 75 °C for a further 1.5 h. The resulting mixture was washed three times with distilled water and ethanol and dried under vacuum (**Scheme S1**).

S1.3. Preparation of Fe_3_O_4_-wax composite microsphere

Following the theory of Pickering emulsions, a homogeneous emulsion of Fe_3_O_4_/wax was first obtained by addition of paraffin wax (5g) to the mixture of well-dispersed SPIONs (300 mg) in ultrapure water (30 mL) under severe mechanical stirring at 78 °C for 1.5 h [2]. Then the homogeneous emulsions were cooled with pouring the mixture in cold water and was washed with water flow and then ethyl alcohol several times to remove the weakly attached magnetic particles and SPION free wax balls, respectively. Finally, the desired Fe_3_O_4_/wax composite microspheres were kept in ethyl alcohol in cold condition (**Scheme S1)**.

S1.4. Modifying waxed SPIONs with APTES

The freshly prepared wax balls (5g) were dispersed in 100 mL of an ethanol solution containing APTES (2.22 mmol, 0.5 mL) and H­_2_O (0.5 mL), then the stirring was continued at room temperature. Silanization of the exposed surface of the SPIONs was done in 8 h. after the completion of the reaction, the mixture was washed with pure ethanol to remove excess amount of APTES, and the Fe_3_O_4_-wax microspheres that are modified with amino groups (APTES- Fe_3_O_4_-wax) were kept in ethanol for further reactions **(Scheme S1)** [2].

*S1.5. Synthesis of poly(ε-caprolactone) (PCL)*

Poly(ε-caprolactone) was synthesized based on the previously reported procedure with some modifications [2]. Briefly, 3 ml of ε-caprolactone (26.28 mmol) was solved in DMF in a two-necked round bottom flask under the inert condition, then 0.35 mmol DL-malic acid was added to the solution and the temperature was raised gradually up to 80 °C. A catalytic amount of Sn(Oct)_2_ was added and the temperature of the reaction was adjusted at 120 °C and let to stir for 24 hours. The produced crude product was dissolved in a minimum amount of DMF and precipitated in cold water to purify the product. The purification process repeated three times and the precipitate was dried in a vacuum oven at 45 °C for 24 h to produce PCL with 85.4 % yield **(Scheme S3)**.

*S1.6. Acrylation of PCL*

The synthesized PCL (12g, 1.51 mmol) and triethylamine (1.11 mL, 8 mmol) were dissolved in 6 mL DMF at 0 °C. Acryloyl chloride (0.48 mL, 6 mmol) was then added dropwise to the mixture, and the reaction was continued at room temperature for 48 hours under dry nitrogen atmosphere. Afterward, to precipitate the acrylated-PCL (APCL), the reaction solution was poured into cold water and the precipitate was filtered. The filtered APCL was dried in a vacuum oven at 40 °C overnight to achieve the resulted macromer with 72% yield (**Scheme S4**) [3].

**References:**

1. Secundo F., Carrea, G., Soregaroli C., Varinelli D. & Morrone R. Activity of different Candida antartica lipase B formulations in organic solvents. *Biotechnol. Bioeng.*, **73**. 157-163 (2001); 10.1002/bit.1047.
2. Shaghaghi B., Khoee S. & Bonakdar S. Preparation of multifunctional Janus nanoparticles on the basis of SPIONs as targeted drug delivery system. *Int. J. Pharm.*, **559**, 1-12 (2019); 10.1016/j.ijpharm.2019.01.020.
3. Khoee, S. & Hemati, K. Synthesis of Magnetite/Polyamino-Ester Dendrimer Based on PCL/PEG Amphiphilic Copolymers via Convergent Approach for targeted Diagnosis and Therapy. *Polymer* **54(21)**, 5574-5585 (2013); 10.1016/j.polymer.2013.07.074.

# S2. Additional Data


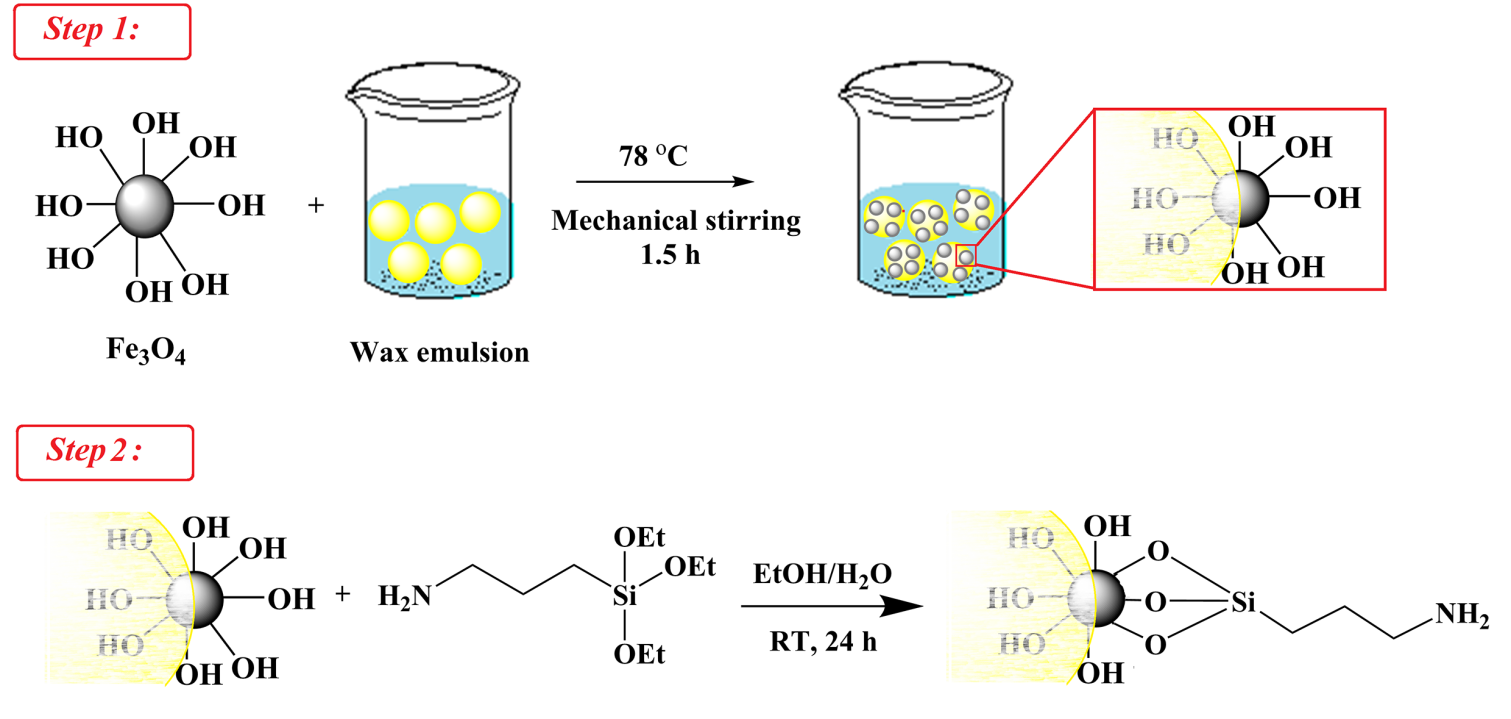


**Scheme S1:** Schematic representations for preparation of Janus microspheres wax-Fe_3_O_4_ (Step 1) and Janus microspheres APTES-Fe_3_O_4_-wax by biphasic grafting at a Pickering emulsion interface (Step 2)


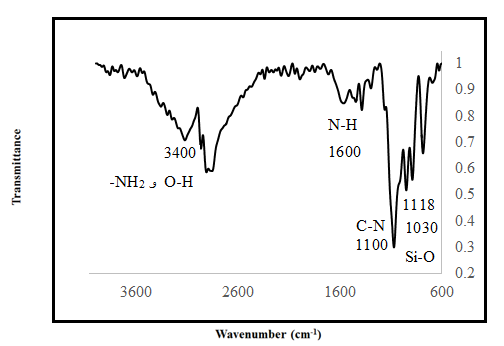


**Figure S1:** FT-IR spectra of NH_2_-Fe_3_O_4_ Janus nanoparticles

**
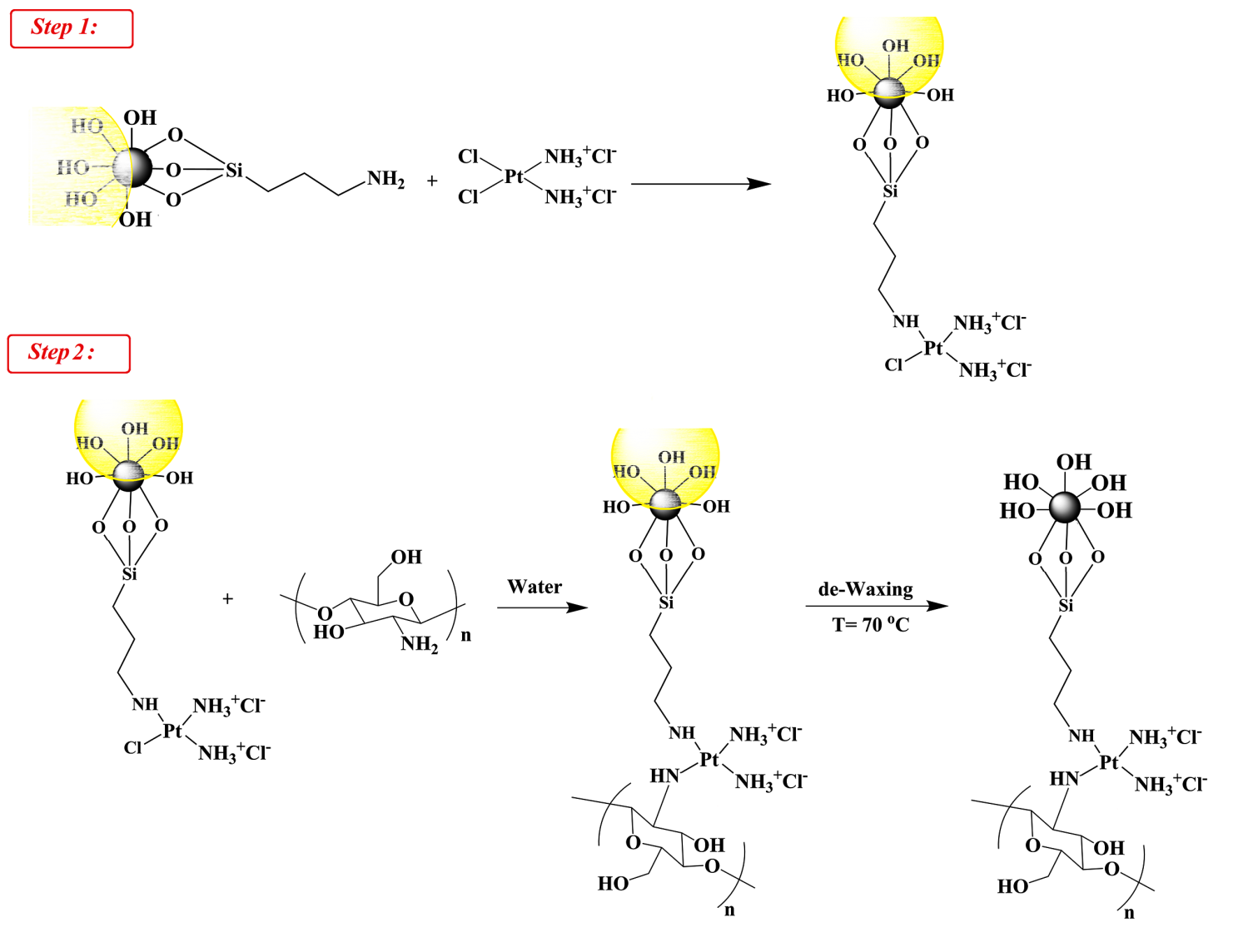
**

**Scheme S2.** Schematic illustration of the preparation of Janus microspheres CisPt-Fe_3_O_4_-wax (Step1) and Janus microspheres CS-CisPt-Fe_3_O_4_-wax via Pickering emulsion (Step2)

**
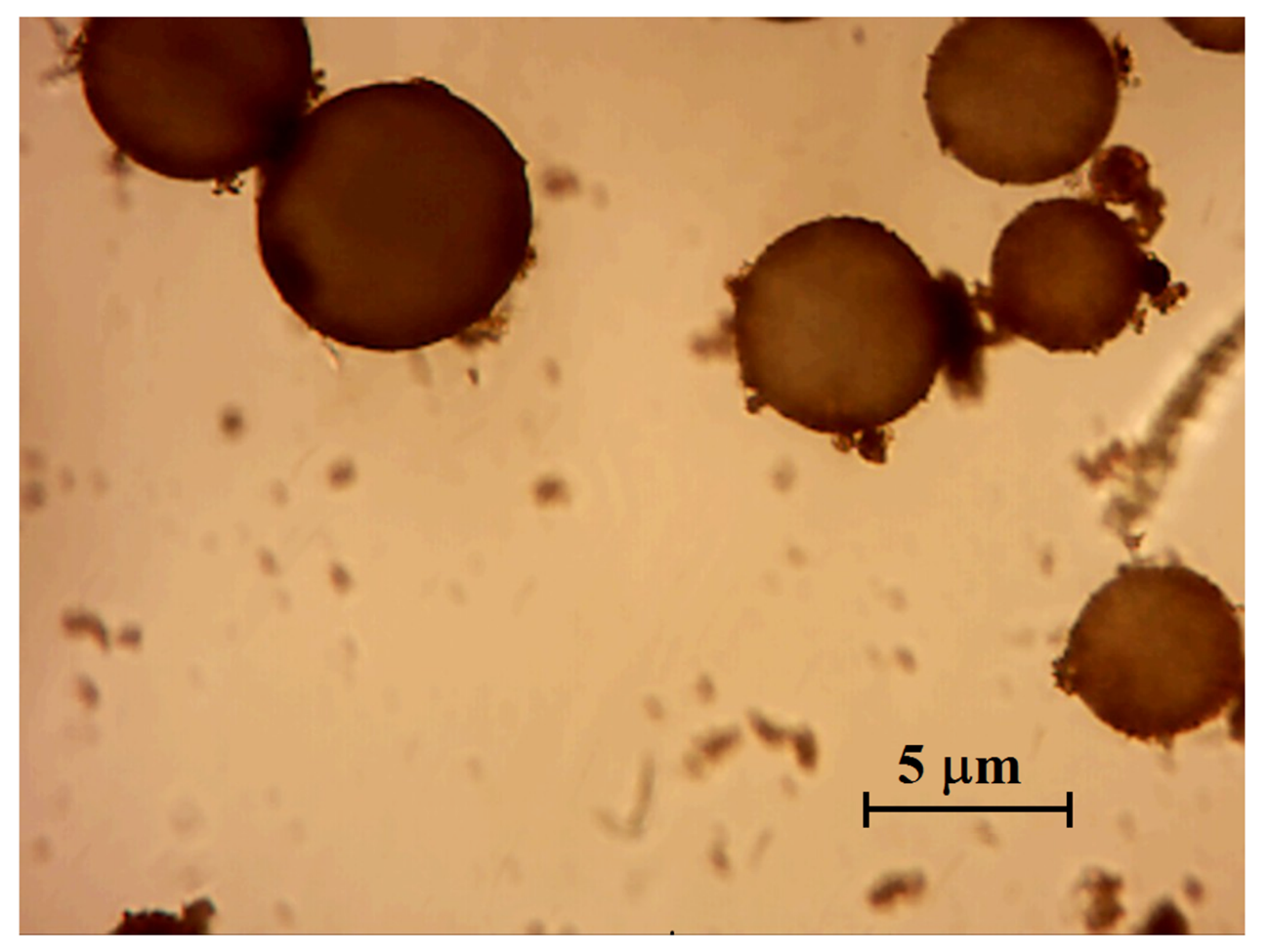
**

**Figure S2.** Optical microscope image of CisPt-Fe_3_O_4_-wax microspheres

**

**

**Scheme S3.** Synthesis of PCL by ring opening polymerization of CL in the presence of malic acid as initiator and tin(II) octoate as catalyst

*
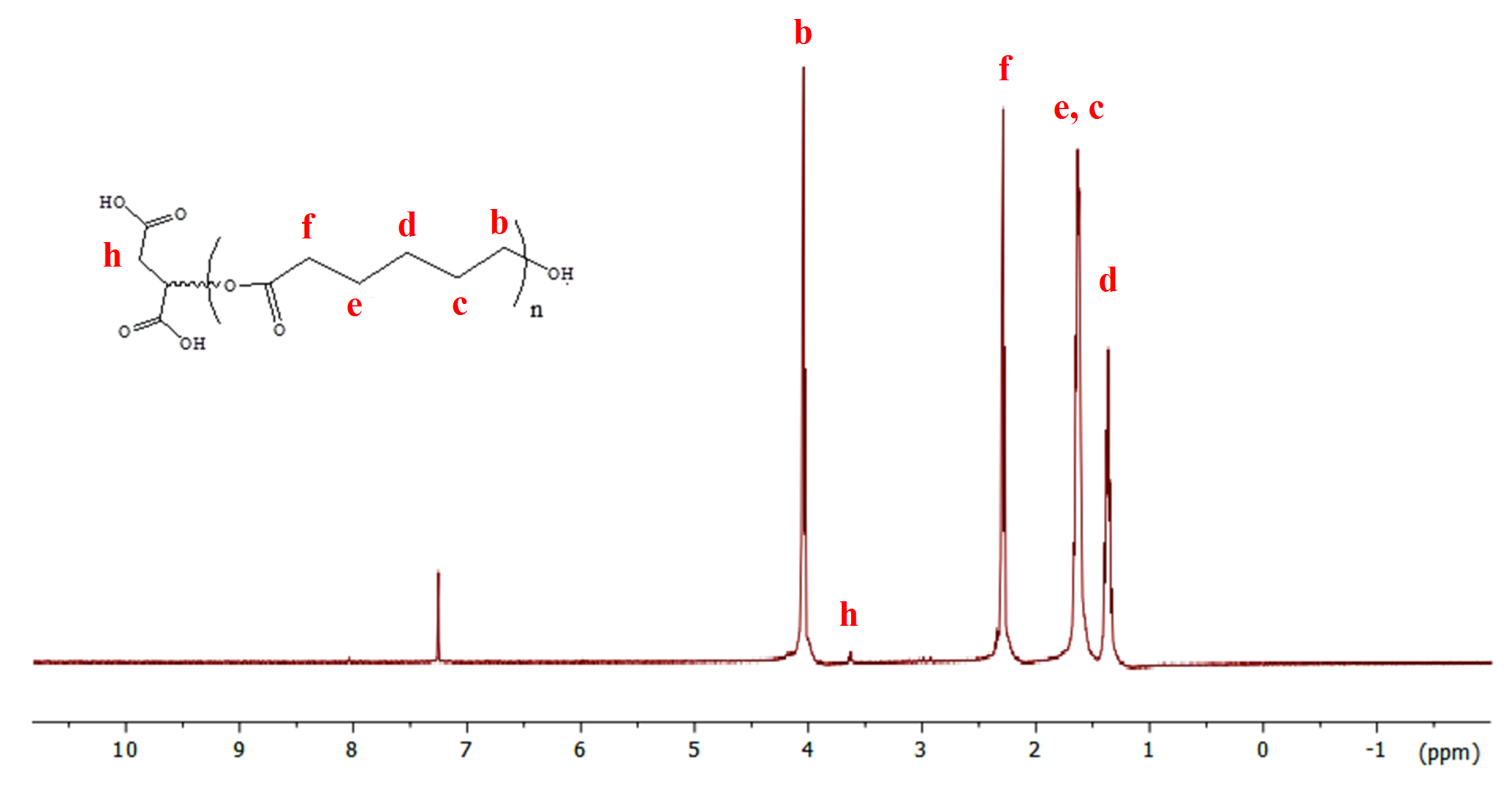
*

**Figure S3:** ^1^H-NMR spectrum of PCL





**Scheme S4.** The synthesis of ACPCL via reaction of PCL with acryloyl chloride and triethylamine


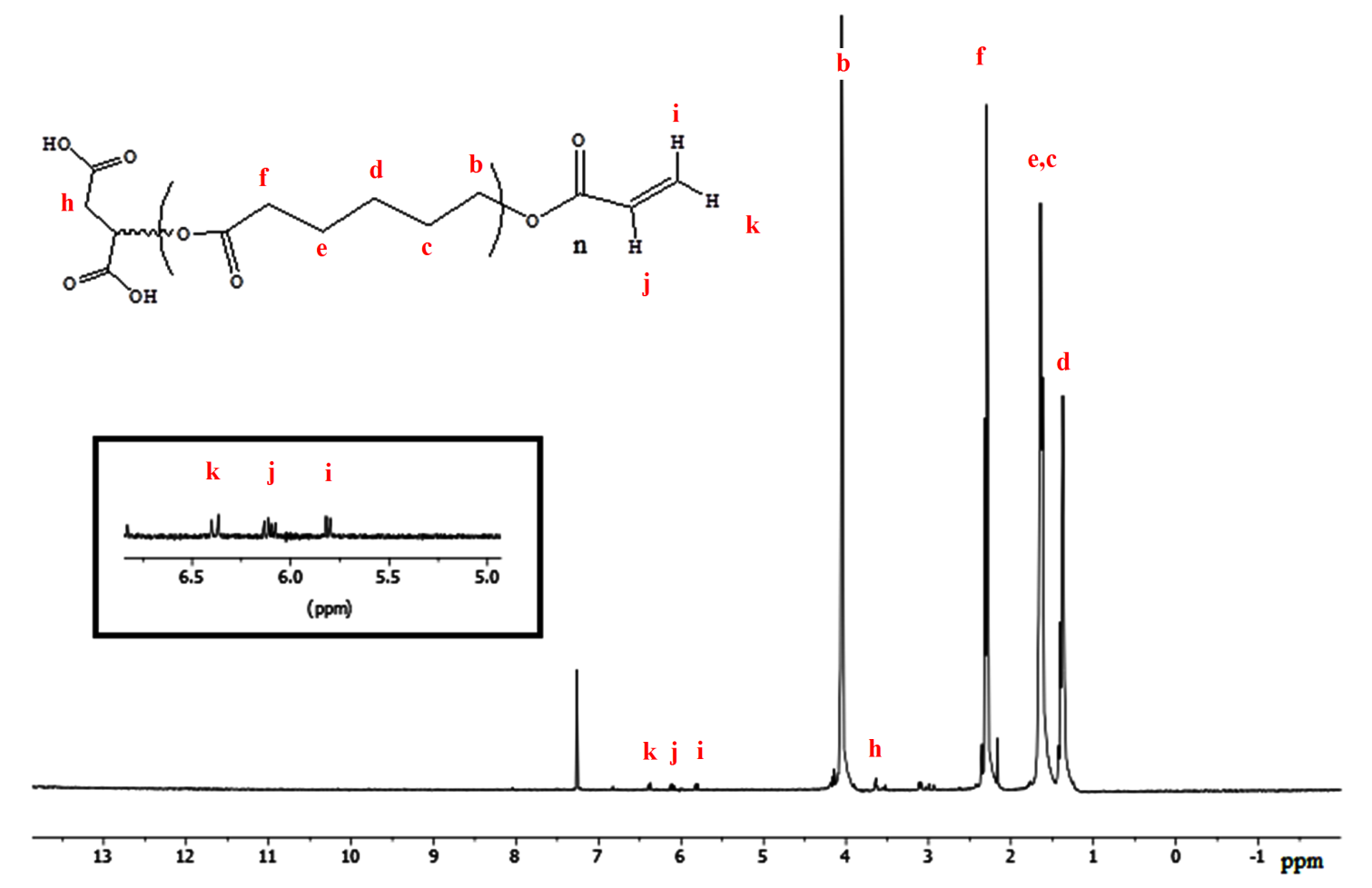


**Figure S4:** ^1^H-NMR spectrum of acrylated PCL (APCL)


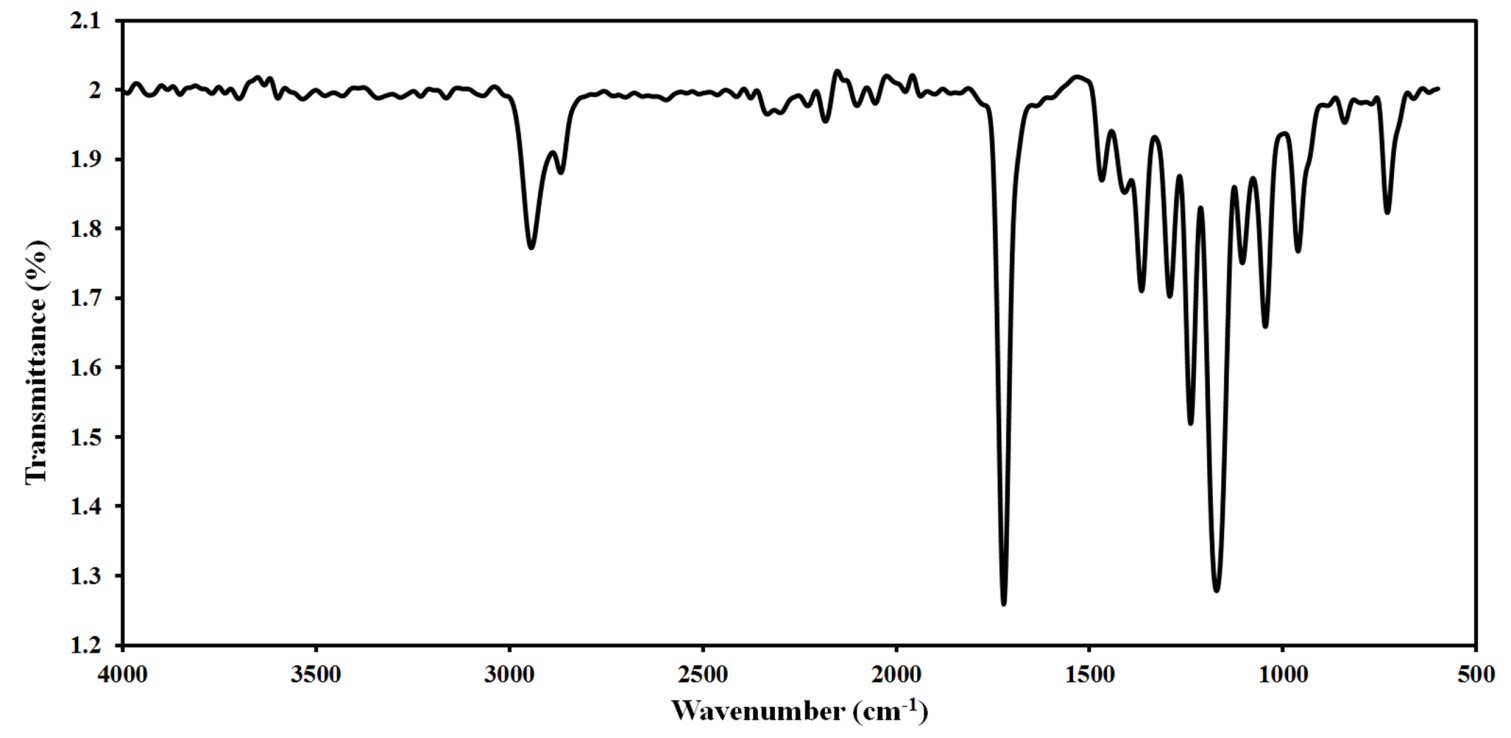


**Figure S5.** FT-IR spectra of acrylated PCL (APCL)

**

**

**Scheme S5.** The synthesis of APAPTS via Michael reaction

**

**

**Scheme S6.** Schematic of the preparation of Cs-CisPt-Fe_3_O_4_-PCL Janus nanoparticle

**Table S1.** Summary of TGA results

| **Sample** | **Water amount (%)** | **1^st^ stage** | | |  | **2^nd^ stage** | | | **Char residue**  **(%)** |
| --- | --- | --- | --- | --- | --- | --- | --- | --- | --- |
|  |  | $\boldsymbol{T}_{\boldsymbol{onset}\boldsymbol{1}}$  **(°C)** | $\boldsymbol{T}_{\boldsymbol{final}\boldsymbol{1}}$  **(°C)** | $\boldsymbol{T}_{\boldsymbol{max}\boldsymbol{1}}$  **(°C)** |  | $\boldsymbol{T}_{\boldsymbol{onset}\boldsymbol{2}}$  **(°C)** | $\boldsymbol{T}_{\boldsymbol{final}\boldsymbol{2}}$  **(°C)** | $\boldsymbol{T}_{\boldsymbol{max}\boldsymbol{2}}$  **(°C)** |  |
| **CS-CisPt-Fe_3_O_4_** | 29.5 | 190 | 358 | 275 |  | - | - | - | 44.06 |
| **J(CS-Fe_3_O_4_-PCL)** | **-** | 180 | 358 | 314 |  | 358 | 464 | 406 | 44.51 |


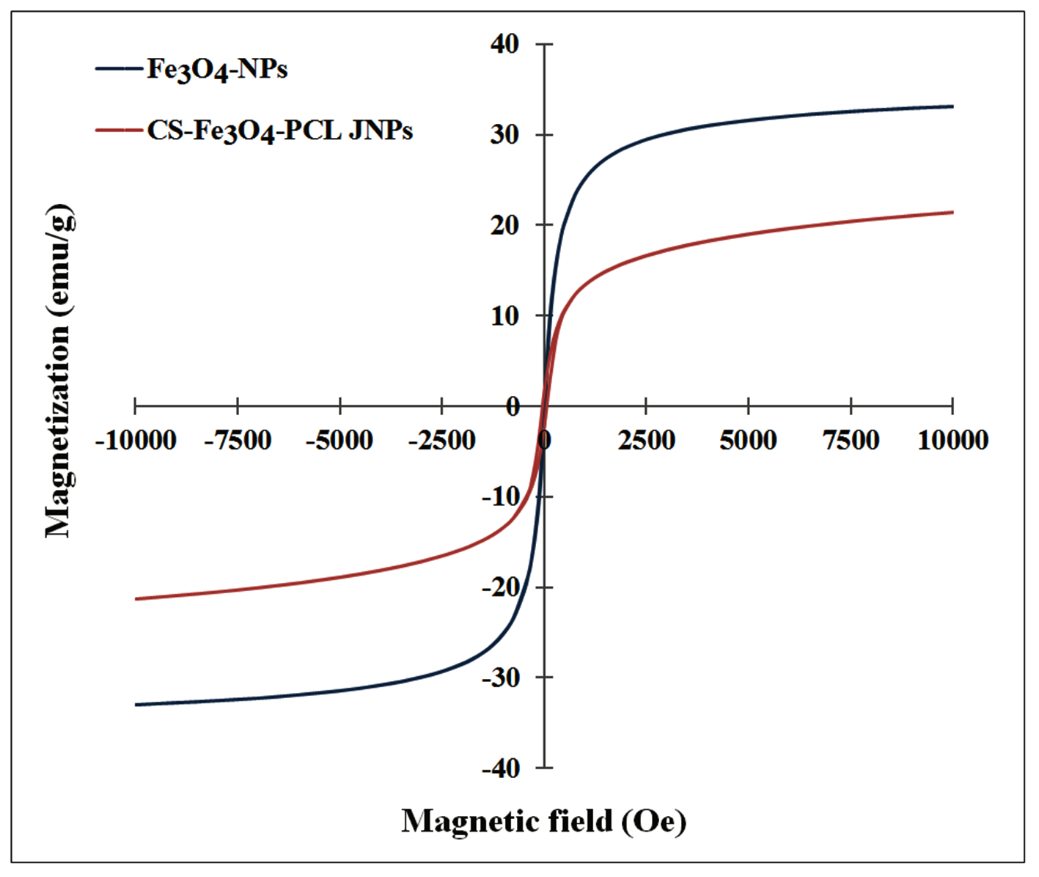


**Figure S6.** Magnetization curves obtained by VSM at room temperature for (a) Fe_3_O_4_, (b) CS-Fe_3_O_4_–PCL JNPs
